# Protocol for a systematic review of the development of depression among adolescents and young adults: psychological, biological, and contextual perspectives around the world

**DOI:** 10.1186/s13643-019-1104-7

**Published:** 2019-07-20

**Authors:** Gloria A. Pedersen, Zuzanna Zajkowska, Christian Kieling, Kamal Gautam, Valeria Mondelli, Helen L. Fisher, Johnna R. Swartz, Abiodun Adewuya, Rakesh Karmacharya, Brandon A. Kohrt

**Affiliations:** 1 School of Medicine and Health Sciences, Division of Global Mental Health, 2120 L St NW, Ste 600, Washington DC, 20037 USA; 2 Institute of Psychiatry, Psychology & Neuroscience, King’s College London, 16 De Crespigny Park, London, SE5 8AF UK; 3 Department of Psychiatry and Child & Adolescent Psychiatry Division, Universidade Federal do Rio Grande do Sul, Hospital de Clínicas de Porto Alegre, Rua Ramiro Barcelos, 2350 – 400N, Porto Alegre, RS 90035-903 Brazil; 4 Transcultural Psychosocial Organization Nepal (TPO Nepal), Anek Marga, Baluwatar, G.P.O. Box 8974/CPC 612, Kathmandu, Nepal; 5 Department of Human Ecology, University of California Davis, One Shields Ave, Davis, CA 95616 USA; 6 Department of Behavioral Medicine, Lagos State University College of Medicine, 1-5 Oba Akinjobi Way, G.R.A., Ikeja P.M.B, Lagos, 21266 Nigeria; 7 Center for Human Genetic Research, Massachusetts General Hospital, 185 Cambridge Street, Boston, MA 02114 USA

**Keywords:** Adolescent, Young adult, Depression, Risk factors, Protective factors, Early diagnosis, Developing countries, Neurosciences, Review, Risk assessment

## Abstract

**Background:**

Depression is a leading contributor to disability-adjusted life-years because of early onset and chronicity throughout the lifecycle. It is crucial to identify early predictors of depression among adolescents and young people to effectively target prevention. A gap in the literature is a comprehensive systematic review of predictors of depression among adolescents around the globe, especially in low- and middle-income countries LMICs. This review aims to identify evidence for biological, psychological, and contextual risk factors for the development of depression among adolescents and young adults (10–24 years of age) in high-income countries (HICs) and LMICs, ultimately contributing to (a) identification of potential mechanisms underlying depression development, (b) selection of common risk and protective factors as targets for detection, and (c) refinement of risk models that can be evaluated through existing cohorts in HICs and LMICs.

**Methods:**

This review will follow the Population, Exposure, Comparison, Outcome (PI(E)CO) model and adheres to the PRISMA-P guidelines. A search strategy was developed by a multidisciplinary research consortium. Seven databases (MEDLINE via Ovid, PsycINFO, Cochrane Database of Systematic Reviews, Web of Science, Lilacs, African Journals Online, Global Health) will be searched to identify articles. Independent raters will screen and retrieve articles for inclusion, conduct quality ratings, and extract data. The Systematic Assessment of Quality in Observational Research adapted for Cultural Psychiatry Epidemiology (SAQOR-CPE) will be used to assess quality of observational studies. We will assess for publication bias using funnel plots and statistical methods. We will use narrative synthesis to present results, addressing the study’s objectives following the Cochrane Handbook guidelines. Meta-analyses will be used to report summary statistics for association of risk factors with development of depression.

**Discussion:**

This systematic review will summarize evidence-based research that examines the psychological, biological, and contextual factors contributing to the onset of depression in adolescents across the globe. Results will support the development of a model that can be evaluated in existing cohorts around the world.

**Systematic review registration:**

PROSPERO registration CRD42018103973.

**Electronic supplementary material:**

The online version of this article (10.1186/s13643-019-1104-7) contains supplementary material, which is available to authorized users.

## Background

The onset for most mental illnesses is during adolescence and early adulthood [1, 2]. This burden of adolescent mental illness is disproportionately borne by young people in low- and middle-income countries (LMICs) who comprise nearly 90% of the world’s youth and who have the least access to services [3–6]. Moreover, one out of three suicides worldwide occurs among adolescents in LMICs [7]. Among mental illnesses, depression is the leading contributor to disability-adjusted life years—in part because of early-onset and chronicity throughout the lifecycle [8]. Therefore, there is a crucial public health need to identify early predictors of depression among adolescents and young people, with special attention to LMICs given the greatest burden and least research in these settings [4]. One key gap in the literature is a systematic review of the predictors of depression among adolescents and young people focusing on the global literature. Therefore, this systematic review will focus on early-life risk and protective factors for depression including studies from both high-income countries (HICs) and LMICs.

Previous reviews have been conducted on risk factors for adolescent depression, although currently, the reviews are focused on specific risk factors and overall generalizability of the evidence is limited, with most reviews only able to report on HICs. For example, Kohler and colleagues conducted an umbrella review mapping risk factors for depression over the lifespan, but with a focus on only environmental factors [9]. Cairns et al. [10] conducted a systematic review on risk and protective factors for adolescent depression, but, focusing only on modifiable factors, the review was not able to address contextual factors such as those at the community or family level. In the same review, of the 113 included studies, 97% of them reported on populations from HICs, a trend found in similar reviews [11, 12]. Moreover, when considering a global perspective on the development of depression, it is important to consider a wider range for the age group of interest (e.g., youth under the age of 25 years old) to account for variance in contextual factors that may play a role in the development of the adolescent brain [13, 14], particularly in LMICs. Currently, of the 12 adolescent systematic reviews found relevant to this review’s topic, only 1 included ages 25 and under [15]; all others included ages 19 years or younger [10–12, 16–24].

Upon completion of this systematic review, we hope to address the above gaps in the literature, as well as contribute to the following endeavors:

- Identify common risk and protective factors across global studies as potential mechanisms underlying the development of depression in adolescence;
- Identify common risk factors as potential targets for early detection and interventions;
- Identify regional and other potential population-specific variation in risk pathways for depression among adolescents as contextual modifiers for the focus of research and intervention adaptation;
- Identify common risk and protective factors to refine predictive models that can be evaluated using existing cohorts in HICs as well as in LMICs.

## Objectives

The primary objective of this review is to identify the evidence for biological, psychological, and contextual risk factors for the development of depression among adolescents (25 years and younger) in HICs and LMICs. The secondary objectives are to identify the evidence for risk and protective factors specifically found within prospective longitudinal observational studies in HICs and LMICs, to identify the evidence for risk and protective factors specifically found within cross-sectional studies conducted in LMICs, and to identify the evidence for a relationship of biological and contextual risk factors with depression in adolescents in HICs and LMICs.

## Methods

This protocol was developed in accordance with the Preferred Reporting Items for Systematic Review and Meta-Analysis Protocols (PRISMA-P) 2015 checklist [25]. This protocol has been registered in the International Prospective Register of Systematic Reviews (PROSPERO), study protocol registration CRD42018103973.

### Operationalization of key constructs

#### Adolescence

The World Health Organization (WHO) recognizes persons aged 10–19 as adolescents and those aged 15–24 as youth, with a comprehensive category of young people referring to ages 10–24 years [13]. Persons between the ages of 10–24 may be defined as adolescents as it is noted that youth changes with circumstances, especially when considering changes in settings demographically, financially, economically, and socio-culturally [14]. Therefore, for the purposes of this review, we will follow the WHO categorization of young people as 10–24 years of age, which is inclusive of adolescence [26].

#### Biomarker

We operationalize biomarker as “a biological characteristic objectively measured and evaluated for indications of normal biological or pathological processes, or a response to a therapeutic intervention” [27].

#### Depression

Usually defined as a common mental disorder, depression is characterized by persistent sadness (or irritability in the case of adolescents) and a loss of interest in activities, accompanied by an inability to carry out daily activities. Symptoms range from mild to moderate and may include (but are not limited to) several of the following: loss of energy, change in appetite, anxiety, restlessness, feelings of worthlessness, guilt, hopelessness, indecisiveness, sleeping more or less, or thoughts of self-harm or suicide. Depression is globally experienced on a continuum [28, 29] and for the purposes of this review will be defined by clinical diagnoses, structured clinical interviews, self-report rating scales, or medical records. The supplemental file includes a list of diagnoses and associated codes meeting the criteria for inclusion for the Diagnostic and Statistical Manual of Mental Disorders and International Classification of Disease (see Additional file 1).

#### Low- and middle-income countries

Low-income economies are $1025 or less gross national income (GNI) per capita in 2015; lower middle-income economies are those with a GNI per capita between $1026 and $4035; upper middle-income economies are those with a GNI per capita between $4036 and $12,475; and high-income economies are those with a GNI per capita of $12,476 or more (https://blogs.worldbank.org/opendata/new-country-classifications-2016). For countries that have changed economic status during the period of longitudinal data collection, we will require that the country met the World Bank criteria for an LMIC lending category at the time of initiating the cohort.

#### Mechanisms of action/mechanisms of change

This review will use the National Institute of Mental Health (NIMH) definition of mechanism of action as a “target of engagement” which may create the potential for development (“go” outcome) or reject this potential (“no-go” outcome) and its ability to modify disease, behavior, or functional outcomes; in other words, it is the basis for the effect and may also be the process or steps responsible for a therapeutic outcome [30–32].

#### Mediators and moderators

A mediator is operationalized as an intervening variable which lies on the causal pathway between the exposure and outcome; it may provide additional information as to how a change or outcome came about, but it is not necessarily a mechanism for change [30, 33]. A moderator is operationalized as a characteristic that influences the direction or magnitude of the relationship between an independent and dependent variable [30].

#### Protective and risk factors

Using Cairns and colleagues’ definitions (p. 63), we will operationalize a protective factor as “an antecedent condition associated with a decrease in the likelihood of the outcome of interest” [10, 34] and a risk factor as “an antecedent condition associated with an increase in the likelihood of the outcome of interest” [10, 34].

### Study eligibility criteria

#### Types of studies

For the initial search of risk and protective factors for depression in adolescence in HICs and LMICs, studies using longitudinal prospective designs will be identified. A second search will focus specifically on LMICs to examine cross-sectional study designs due to the lack of longitudinal studies evidenced in these settings. Finally, when considering the relationship between biological, psychological, and contextual factors and adolescent depression, we will include longitudinal and cross-sectional studies and intervention and prevention trials in order to capture any biological or contextual factors that may be evidenced while considering the gap in longitudinal study designs conducted in LMICs. Trials or experimental designs will only be included when examining biological factors in order to expand the pool of potential studies. Discussion papers, letters, editorials, case studies or case series, and qualitative studies without a quantitative element will be excluded.

#### Type of participants and settings

Both sexes in adolescent populations will be included in this review. Included studies will require a depression outcome (as defined by a categorical DSM/ICD diagnosis, clinical diagnosis, medical records, structured interview, or self-report measure). Eligible populations will be young people in high-, middle-, and low-income countries who are under the age of 25 years at study baseline and have been evaluated for depression with at least one subsequent time point of at least 6 months. The LMIC-specific analysis will also include young people living in LMICs who are under the age of 25 years who have been evaluated for the presence of depression or depressive symptoms. The biological factors’ objective will include adolescents in high-, middle-, and low-income countries who are under the age of 25 years and were evaluated for depression and for biological markers of interest, as well as psychological or contextual markers associated with depression. Given the high rates of psychiatric co-morbidity, populations with depression plus other psychiatric conditions (including substance abuse) will be included for the risk and protecte factors part of the review. Studies limited only to specific medical subpopulations (e.g., only young people with HIV, diabetes, intellectual disabilities) that do not include comparative populations will be excluded. Any longitudinal studies with an initial prospective time point of under 6 months will also be excluded. Medication or other treatment status will not be an exclusion criterion, but information will be reported on these whenever available.

#### Type of exposures

The following factors will be evaluated for association with depression among young people:

- Contextual factors associated with an increased risk for developing major depression during adolescence (this review will include demographic, economic, family, neighborhood, environmental, and social and cultural factors; in addition, we will explore life events and traumatic exposures);
- Biological markers associated with an increased risk for developing major depression during adolescence. This review will focus on studies that address the following: hypothalamic-pituitary-adrenal axis (HPA axis), inflammation, endocannabinoids, vitamins, polyunsaturated fatty acids (PUFAs), hormones, neurotrophic factor, neurotransmitters, telomere/gene length, neuroplasticity, and gene expression (including mRNA quantification);
- Brain-related abnormalities in individuals at risk for major depression during adolescence (this review will focus on magnetic resonance imaging studies, both structural and functional, assessing children/adolescents at risk);
- Psychological factors associated with increased or decreased risk for developing major depression during adolescence (this review will include factors such as self-esteem, locus of control, cognitive biases, emotional regulation, reward responsivity, and learned helplessness).

### Comparators

Comparison groups will vary between objectives (see Appendix 1) but will broadly include adolescents who are not exposed to the factor of interest prior to the time of assessment or adolescents who do not develop depression during the assessed periods of adolescence and young adult development.

### Outcomes

The primary outcome will be development of depression among young people with a cutoff of 25 years of age or younger and also allowing for studies that include earlier endpoints of depression development, in any population, i.e., high-, middle-, and low-income countries. The primary outcome measures of depression may include self-reports, adult-informant reports, structured observations in clinical settings, and clinical records.

#### Timing of outcome assessment

Eligible studies should include assessment of depression during at least one time period between the ages of 10 and 24 years and a follow-up time point at least 6 months later.

### Search strategy for identifying relevant studies

We will search the following electronic databases to identify potentially relevant studies for our review: MEDLINE (via Ovid), PsycINFO, Cochrane Database of Systematic Reviews, Web of Science (Core Collection), Lilacs, African Journals Online, and Global Health. In addition to these electronic databases, we also plan to identify relevant studies by reviewing reference lists of eligible studies and review articles and by contacting experts and authors of eligible studies by email. Relevant systematic reviews from the database outputs will be hand-searched for any eligible studies which may have been missed. Only published research in academic journals will be used for objectives 1 and 3 of this review (see below). Searches will be conducted in English, though publication language will not be a limiter. The Boolean operator “OR” will be used to find articles with one or more search terms and synonyms and “AND” to combine the different concepts for relevant articles. Where applicable, MeSH subheadings will be used, and “keywords” where relevant for given databases. For objective 2 (see below), which will use data from LMIC, we will expand our search to grey literature. Some studies of adolescents in LMIC are presented in reports of nongovernmental organizations, and these findings may not be reported in the academic literature. Therefore, we will review reports from international and national nongovernmental organizations and will attempt to identify additional literature through listservs used by mental health psychosocial practitioners working in nongovernmental organizations.

We will use truncation and wildcards to account for UK and US spelling and terminology and abbreviation. The concept combinations will vary with objective (see Appendix 1), but the three main concepts combined will include Population AND Exposure AND Outcome, for example, adolescence AND risk/protective factors AND depression. Years of publication will not be used to limit the search output, but extraction will begin in descending chronological order, assessed at the first 10 years for sufficient robust evidence, then proceed to the next 10 years or more if deemed necessary to capture representative data.

Searches will be re-run before the date of final analyses to identify recent publications in the field.

Objective 1—identify risk and protective factors associated with the development of depression among adolescents and young people in populations evaluated for at least 6 months (i.e., longitudinal samples).

Sample MEDLINE (Ovid) search:

Adolescent/ OR “adolesc*” AND Depression/ OR Depressive disorder/ AND Longitudinal Studies/ OR “longitudinal”

Objective 2—identify risk and protective factors associated with depression among adolescents and young people in LMICs.

Sample MEDLINE (Ovid) search:

Adolescent/ OR “adolesc*” AND Depression/ OR Depressive disorder/ AND Developing Countries/ OR “developing countr*” OR World Bank List (as keywords)

Objective 3—identify risk and protective factors associated with biological markers, brain related abnormalities, and context among adolescents and young people.

Sample MEDLINE (Ovid) search:

Adolescent/ OR “adolesc*” AND Depression/ OR Depressive disorder/ AND “HPA axis” OR Pituitary-Adrenal System

See Appendix 1 for full MEDLINE search, Objective 1.

### Data collection

#### Selection of studies

A charting form will be used to identify studies to be included in the review according to the inclusion and exclusion criteria. Two review authors will independently review titles and abstracts of studies identified through the search strategy and those from additional resources (e.g., contacting authors and searching reference lists of included papers, which will be done through hand searches, Web of Science citation records, or Scopus when appropriate) to determine whether studies potentially meet the inclusion criteria. To assess for study eligibility, two reviewers will independently review the full publication texts of studies identified through title and abstract review. They will record justification for exclusion for each excluded study. A third author will resolve disagreements between the two reviewers.

#### Retrieval of studies

The full text of the selected articles will be retrieved. When a full article is not available through the database or university library services, it will be searched through ResearchGate and Google Scholar, or a request to share the full article will be sent by email to the corresponding author. After the request has been sent, a reminder will be sent after a week. If there is no response within 2 weeks from the reminder, this document will be excluded from the systematic review.

#### Data extraction and management/coding

Extraction fields will include, but not be limited to, article information (publication year, journal), study characteristics (country, urban vs. rural setting, funding), population characteristics (age at enrolment, current age, number of longitudinal assessments), depression assessment (depression criteria, duration of depression, assessment tool, co-morbidity), risk factors (poverty, trauma, family history), protective factors (education, social capital), assessment tool (instrument names and psychometric properties for depression, risk, and protective factors), and biological variables (interleukins, cortisol, galvanic skin response, neuroimaging). Two authors will independently extract data from the published reports of included studies using a standardized, electronic form (e.g., in Microsoft Excel), which will be developed and pre-piloted to assure interrater agreement. We will attempt to collect missing information by contacting the corresponding authors via email.

### Assessment of risk of bias in included studies

Two reviewers will independently assess the risk of bias in included studies by evaluating the following domains:

- Recruitment and sampling strategy
- Follow-up strategy, retention, and selective attrition
- Completeness of outcome data
- Validity and other psychometrics of depression outcomes and other tools used
- Selective outcome reporting
- Other sources of bias related to context, procedures, and reporting

We will assess the quality of observational studies using the Systematic Assessment of Quality in Observational Research (SAQOR) [35] and the adapted SAQOR Cultural Psychiatry Epidemiology (SAQOR-CPE) [36]. If any intervention studies are included, we will limit the analyses to be pre-treatment findings or control groups. We will apply the SAQOR-CPE to the samples of interest in the intervention studies. Quality level will be recorded as high, moderate, low, or very low risk of bias for each domain, and bias rating will be compared between the two author reviewers. A third author will resolve discrepancies. Emails to authors will be used to obtain additional information to reduce sources of bias and adjust risk.

### Data analysis

#### Measurement of exposure effect

The primary comparison for this systematic review will be development of depression before the age of 25 years old. We anticipate that there will be substantial heterogeneity between studies (including reporting differences) that may limit our ability to conduct quantitative syntheses of findings. Similarly, we anticipate heterogeneity in measurement of depression and depressive symptoms, as well as validation, evaluation, and translation of tools. Where there is adequate homogeneity, we will synthesize results using random-effects meta-analysis. We will use standardized mean differences (SMDs) for continuous outcomes and risk ratios for binary outcomes and calculate 95% confidence intervals and two-sided *p* values for each outcome. When applicable, we will use SMDs to generate pooled estimates to compare different types of risk factors. We will conduct all quantitative tests using appropriate techniques in Stata, R, and in Comprehensive Meta-Analysis (Biostat, Englewood, NJ, https://www.meta-analysis.com/). We will follow Ioannidis and colleagues [37] recommendations for conducting meta-analyses and reporting summary statistics. Rather than a priori excluding use of meta-analysis or setting specific criteria for degree of heterogeneity, Ioannidis and colleagues state that it is preferable to present the summary statistic, while clearly documenting the heterogeneity and its possible causes. We employ methods recommended by Ioannidis and colleagues to report and adjust for heterogeneity.

#### Unit of analysis issues

The study population will be the unit of analysis. We do not anticipate obtaining sufficient access to individual-level data to conduct individual-level analyses. Studies that only include cluster-level analysis will not be included.

#### Dealing with missing data

For missing measures of precision (confidence intervals or standard deviations) that remain unavailable after attempting to contact study authors, we will assume the highest variability observed within the group of studies being analyzed.

#### Assessment of reporting bias

We will assess for publication bias using funnel plots. In addition, we will use the approach outlined by Begg and Mazumdar [38] to statistically test for publication bias.

#### Data synthesis

We will provide a narrative synthesis of findings from the included studies. Following Cochrane guidelines [39], we will provide narrative synthesis information separately by study type. From the studies, we develop a conceptual framework to identify themes. This will be done at the full-text stage after reviewing the manuscripts. We will provide descriptives, compare studies on characteristics, tabulate results to identify patterns across studies, use vote counting, and translate data for thematic and content analyses per Cochrane recommendations. For quantitative synthesis, we will provide summaries of the primary outcome measures for each study. We will include forest plots summarizing results of individual studies and the meta-analyses. Summaries of depression outcomes will be subcategorized accordingly by self-report, parent or teacher report, clinical instrument, or healthcare service diagnosis. Also, specifically for the biological factors, summaries will be stratified by those with and without substance abuse diagnoses. We will run meta-analyses for each objective with meta-regression where appropriate, as well as assess methodological issues and conduct sensitivity analyses.

### Subgroup analysis and assessment of heterogeneity

If possible, we will conduct subgroup analyses based on HICs vs. LMICs, by age of onset, co-morbidities, outcome type (e.g., self-report, clinical interview, medical records), geographic region, and population settings (primary care, non-psychiatric specialty services, communities, religious centers, schools). Some included studies may report onset of depression after 25 years of age for some participants. Therefore, we will potentially conduct subgroup analyses by age group of onset: those who have developed depression before the age of 25 and those who have developed it after the age of 25 for studies that follow participants into adulthood. Subgroup analyses will be conducted in Comprehensive Meta-Analysis, including analysis of statistical difference among subgroups. We will assess heterogeneity using the chi-squared test, *I*-squared statistic, and prediction interval [40]. We will evaluate for potential sources of heterogeneity. We will address heterogeneity by using random-effects meta-analysis models and sensitivity analyses. We will use the approach of referring to “low, moderate, and high” heterogeneity at the *I*^*2*^ thresholds of 25%, 50%, and 75%, with the caveat that quantification of heterogeneity will be considered only one marker of variability across studies [41] and the concerns raised by Cuijpers [42] that even point estimates of *I*^*2*^ may have wide confidence intervals extending into the ranges of high heterogeneity.

### Sensitivity analysis

We will conduct sensitivity analyses as recommended by the Cochrane Handbook (Section 9.7) [43]. For example, sensitivity analyses will be conducted removing the studies with the strongest effects sizes and studies with the largest sample sizes. We will follow the approach described in Patsopoulos et al. [44] of excluding up to two studies to see if heterogeneity crosses the threshold below 50% or 25%.

### Assessing overall confidence in cumulative evidence

We will use the Confidence in the Evidence from Reviews of Qualitative research (CERQual) tool to assess confidence in the qualitative syntheses. Similarly, we will use SAQOR-CPE in reference to quantitative reviews.

### Presentation of results

We will present the results of our systematic review through a narrative description addressing the study’s objectives and through charts and tables as recommended in the Cochrane Handbook (Section 11) [43]. First, we will tabulate data from our search process into a flowchart indicating the number of studies identified and excluded at key steps, according to the PRISMA guidelines [25]. Second, we will summarize information on individual studies in a “Characteristics of Included Studies” table. Third, we will present “Data and Analyses” tables and forest plots to present outcome data from individual studies and from any meta-analyses. Fourth, we will present a “Summary of Findings” table to summarize key data available, magnitude of effect of the risk and protective factors studied, and quality of evidence addressing the key study objectives.

## Discussion

There is a crucial public health need to identify early predictors of depression among adolescents and young people around the globe, with special attention to LMICs given that the greatest burden and least research is found in these settings. This systematic review aims to address gaps in the review literature and will summarize evidence-based research which looks at the psychological, biological, and contextual factors contributing to the onset of depression in adolescents and young people across the globe. Recommendations will be made to inform future research priorities. The results will support the exploration of the feasibility and acceptability of early detection and interventions in LMICs and support the development of a common risk factor assessment model that can be evaluated using existing cohorts in HICs as well as in LMICs. These results will inform relevant academic and practitioner communities, key stakeholders such as policy- and decision-makers, and adolescents and their families.

### Additional file

Additional file 1: List of diagnoses and associated codes meeting the criteria for inclusion for the Diagnostic and Statistical Manual of Mental Disorders and International Classification of Disease. (DOCX 12 kb)

## Data Availability

Not applicable.

## Appendix 1

**Table 1:** MEDLINE (Ovid) search, objective 1

1. ADOLESCENT/	
2. (adolesc* or "youth" or "teen" or "young people" or "young person*").mp. [mp=title, abstract, original title, name of substance word, subject heading word, floating sub-heading word, keyword heading word, protocol supplementary concept word, rare disease supplementary concept word, unique identifier, synonyms]	
3. Young Adult/	
4. 1 or 2 or 3	
5. DEPRESSION/	
6. Depressive Disorder/	
7. Depressive Disorder, Major/	
8. ("depressive disorder" or "symptoms of depression" or "major depressive disorder").mp. [mp=title, abstract, original title, name of substance word, subject heading word, floating sub-heading word, keyword heading word, protocol supplementary concept word, rare disease supplementary concept word, unique identifier, synonyms]	
9. 5 or 6 or 7 or 8	
10. LONGITUDINAL STUDIES/	
11. Prospective Studies/	
12. Observational Study/	
13. (longitudinal or prospective or "observational stud*").mp. [mp=title, abstract, original title, name of substance word, subject heading word, floating sub-heading word, keyword heading word, protocol supplementary concept word, rare disease supplementary concept word, unique identifier, synonyms]	
14. 10 or 11 or 12 or 13	
15. 4 and 9 and 14	

## Abbreviations

DSM: Diagnostic and Statistical Manual of Mental Disorders
GNI: Gross national income
HIC: High-income country
HPA axis: Hypothalamic-pituitary-adrenal axis
ICD: International Classification of Diseases
LMIC: Low- and middle-income countries
NIMH: National Institutes of Mental Health
PI(E)COS: Population, Exposure, Comparators, Outcomes, and Study designs
PRISMA: Preferred Reporting Items for Systematic Reviews and Meta-Analyses
PRISMA-P: Preferred Reporting Items for Systematic Review and Meta-analysis Protocols
PROSPERO: International Prospective Register of Systematic Reviews
PUFAs: Polyunsaturated fatty acids
RNA: Ribonucleic acid
SAQOR: Systematic Assessment of Quality in Observational Research
SAQOR-CPE: Systematic Assessment of Quality in Observational Research—Cultural Psychiatry Epidemiology
SMD: Standardized mean differences
WHO: World Health Organization
