# Supplementary material for: Protocol for a systematic review of the development of depression among adolescents and young adults: psychological, biological, and contextual perspectives around the world
Source: Syst Rev. 2019 Jul 20;8:179. doi: 10.1186/s13643-019-1104-7 (PMC6642734; doi:10.1186/s13643-019-1104-7)
Supplement: Supplementary file 1 — List of diagnoses and associated codes meeting the criteria for inclusion for the Diagnostic and Statistical Manual of Mental Disorders and International Classification of Disease. (DOCX 12 kb) [file 13643_2019_1104_MOESM1_ESM.docx]

**Diagnoses eligible for inclusion**

When clinical diagnoses are provided, the following DSM-5 and ICD-11 diagnoses are considered eligible. Comparable diagnoses are acceptable for DSM-IV and ICD-10.

**DSM5 Codes and Diagnoses**

296.36 F33.42 Major depressive disorder, Recurrent episode, In full remission

296.35 F33.41 Major depressive disorder, Recurrent episode, In partial remission

296.31 F33.0 Major depressive disorder, Recurrent episode, Mild

296.32 F33.1 Major depressive disorder, Recurrent episode, Moderate

296.33 F33.2 Major depressive disorder, Recurrent episode, Severe

296.30 F33.9 Major depressive disorder, Recurrent episode, Unspecified

296.34 F33.3 Major depressive disorder, Recurrent episode, With psychotic features

296.26 F32.5 Major depressive disorder, Single episode, In full remission

296.25 F32.4 Major depressive disorder, Single episode, In partial remission

296.21 F32.0 Major depressive disorder, Single episode, Mild

296.22 F32.1 Major depressive disorder, Single episode, Moderate

296.23 F32.2 Major depressive disorder, Single episode, Severe

296.20 F32.9 Major depressive disorder, Single episode, Unspecified

296.24 F32.3 Major depressive disorder, Single episode, With psychotic features

300.4 F34.1 Persistent depressive disorder (dysthymia)

311 F32.9 Unspecified depressive disorder

**ICD-11 Codes and Diagnoses**

6A70 Single episode depressive disorder

6A70.0 Single episode depressive disorder, mild

6A70.1 Single episode depressive disorder, moderate, without psychotic symptoms

6A70.2 Single episode depressive disorder, moderate, with psychotic symptoms

6A70.3 Single episode depressive disorder, severe, without psychotic symptoms

6A70.4 Single episode depressive disorder, severe, with psychotic symptoms

6A70.5 Single episode depressive disorder, unspecified severity

6A70.6 Single episode depressive disorder, currently in partial remission

6A70.7 Single episode depressive disorder, currently in full remission

6A70.Y Other specified single episode depressive disorder

6A70.Z Single episode depressive disorder, unspecified

6A71 Recurrent depressive disorder

6A72 Dysthymic disorder

6A73 Mixed depressive and anxiety disorder

6A7Y Other specified depressive disorders

6A7Z Depressive disorders

SD82 Depression disorder
